# Metabolic Reprogramming in Gut Microbiota Exposed to Polystyrene Microplastics

**DOI:** 10.3390/biomedicines13020446

**Published:** 2025-02-12

**Authors:** Jinhua Chi, Jeffrey S. Patterson, Yan Jin, Kyle Joohyung Kim, Nicole Lalime, Daniella Hawley, Freeman Lewis, Lingjun Li, Xuan Wang, Matthew J. Campen, Julia Yue Cui, Haiwei Gu

**Affiliations:** 1 College of Health Solutions, Arizona State University, Phoenix, AZ 85004, USA; jinhua.chi@asu.edu (J.C.); jspatte5@asu.edu (J.S.P.); lingjun2@asu.edu (L.L.); 2 Center for Translational Science, Florida International University, Port St. Lucie, FL 34987, USA; yjin105@asu.edu; 3 Department of Environmental & Occupational Health Sciences, University of Washington, Seattle, WA 98195, USA; kk1109@uw.edu (K.J.K.); juliacui@uw.edu (J.Y.C.); 4 School of Biological and Health Systems Engineering, Arizona State University, Tempe, AZ 85287, USA; nlalime@asu.edu; 5 School of Life Sciences, Arizona State University, Tempe, AZ 85287, USA; dphawle1@asu.edu (D.H.); wangxuan@asu.edu (X.W.); 6 Environmental Health Sciences, Florida International University, Miami, FL 33199, USA; flewi017@fiu.edu; 7 Department of Pharmaceutical Sciences, College of Pharmacy, University of New Mexico Health Sciences, Albuquerque, NM 87106, USA; mcampen@salud.unm.edu

**Keywords:** gut microbiota, microplastics, mass spectrometry, metabolomics, 16S rRNA

## Abstract

**Background**: Microplastics (MPs) are small plastic fragments with diameters less than 5 mm in size and are prevalent in everyday essentials and consumables. Large global plastic production has now led to a flooding of MPs in our natural environment. Due to their detrimental impacts on the planet’s ecosystems and potentially our health, MPs have emerged as a significant public health concern. In this pilot study, we hypothesize that MPs exposure will negatively affect gut microbiota composition and function, in which metabolic reprogramming plays an important role. **Methods**: Using in vitro experiments, three bacterial strains (*Escherichia coli* MG1655, Nissle 1917, and *Lactobacillus rhamnosus*) were selected to investigate the impacts of MPs exposure. The bacterial strains were individually cultured in an anaerobic chamber and exposed to 1 µm polystyrene MPs at various concentrations (0, 10, 20, 50, 100, and 500 µg/mL) in the culture medium. **Results**: MPs exposure reduced the growth of all three bacterial strains in a dose-dependent manner. Liquid chromatography mass spectrometry (LC-MS)-based untargeted metabolomics revealed significant differences in multiple metabolic pathways, such as sulfur metabolism and amino sugar and nucleotide sugar metabolism. In addition, we extracted gut microbiota from C57BL/6 mice, and 16S rRNA sequencing results showed a significant upregulation of *Lactobacillales* and a significant reduction in *Erysipelotrichales* due to MPs exposure. Furthermore, targeted and untargeted metabolomics corroborated the in vitro results and revealed alterations in microbial tryptophan metabolism and energy producing pathways, such as glycolysis/gluconeogenesis and the pentose phosphate pathway. **Conclusions**: These findings provide evidence that MPs exposure causes comprehensive changes to healthy gut microbiota, which may also provide insights into the mechanistic effects of MPs exposure in humans.

## 1. Introduction

All forms of plastics less than 5 mm but greater than 1 μm in length are typically classified as microplastic particles (MPs) [1]. These particles can be found in a variety of everyday sources, such as cosmetics, clothing, food packaging, and industrial processes [2,3,4,5]. The pervasive presence of MPs is now impacting aquatic and terrestrial ecosystems on a global scale. In 2014, it was estimated that oceans contained as high as fifty trillion MPs with a cumulative weight of 235,000 tons [6]. In fact, recent evidence has shown an infiltration into the earth’s water cycle with the presence of MPs in rain and snow [7]. Global plastic production has also increased nearly 28% in the last five years and may eclipse five hundred million tons in 2024 [6]. MPs have a wide range of half-lives, from thirty to three hundred years, which are mainly dependent upon width and composition, but MPs never completely degrade [8]. Due to this slow rate, MPs continue to persist at high levels in the environment. The exponential rise in global plastic manufacturing, coupled with slow MPs degradation, means such exposures are guaranteed to increase dramatically in the coming decades with plastic waste potentially reaching twelve billion tons by 2050 [9,10,11].

MPs have become an emerging concern due to their ubiquitous presence in nature and their potential detrimental effects on human health. It is estimated that humans ingest nearly 211,000 microplastic particles annually with almost 142,000 particles entirely through dietary consumption [5,12,13]. This chronic ingestion is further observed through their presence in human whole blood and tissue samples [14,15,16]. MPs intake has shown various adverse effects to human health, including mucosal irritation, inflammation, and tissue damage [17,18,19,20]. Although the long-term impact of MPs on health is still not fully understood, current evidence also suggests deleterious impacts on immune, endocrine, and reproductive systems [21,22]. Accumulation of MPs exposure can initiate an immune response in male reproductive glands, resulting in increased expression of inflammatory factors and cytokines [21]. Due to the activation of the central transcription factor, nuclear factor-κB (NF-κB), induced apoptosis of affected cells may occur, leading to distorted reproductive organ morphology [21,23,24]. In addition, MPs are common carriers for toxic chemicals and other pollutants, which have been linked to the development of cancer and other conditions [25]. A study of simulated human gastrointestinal (GI) digestion was able to show that MP exposure resulted in altered colonic community composition and adherence of microbiota to MPs [26]. Recent case studies of MPs and the human gut microbiome reported that the composition, diversity, and functional pathways of gut microbiota were significantly altered, and these alterations persisted after the exposure ended [27,28,29]. While chronic human exposure to MPs is gaining recognition, the mechanism of how it induces a variety of health risks is still largely unknown.

MPs are primarily introduced into the human body through the GI tract, where they eventually enter the circulatory system and reach other tissues [30,31]. The GI tract is host to a plethora of microorganisms, such as bacteria, archaea, viruses, and fungi, which is collectively referred to as the gut microbiome. Typically, six bacterial phyla (*Firmicutes*, *Bacteroidetes*, *Actinobacteria*, *Proteobacteria*, *Fusobacteria*, and *Verrucomicrobia*) mainly comprise the gut microbiome with *Firmicutes* and *Bacteroidetes* being the most prominent microorganisms [32]. The significant impact of these microbiota on health has gained profound interest in recent years. Dysbiosis, an imbalance of the microbiome, has been linked to several GI conditions, such as inflammatory bowel disease (IBD), irritable bowel syndrome (IBS), obesity, and type 2 diabetes (T2D) [33]. Recent studies have also shown the effect of MPs on GI disorder development, including increased intestinal inflammation, oxidative stress, permeability, and intestinal flora ailments [34,35]. Moreover, the interactions between MPs and gut microorganisms interfere with intestinal barrier function, which may lead to malabsorption and greater permeability of pathogens and hormone-disrupting compounds. When ingested, MPs have also shown to carry microbial loads and be vectors of pathogens as well [36]. Interestingly, similar results have also been observed in animal models including mice and zebrafish [37,38]. Studies have shown the presence of MPs in mice tissues throughout the body, as well as MPs-induced dysbiosis and disordered hepatic lipid metabolism [38,39].

The gut microbiome has an essential role in host digestion and absorption of both macro and micronutrients, which indicates its large influence in overall metabolism [40]. In recent years, the field of metabolomics has provided an effective approach to examine these small metabolite molecules within a biological system to offer insights into the metabolic pathways and alterations associated with various conditions [41,42,43,44,45]. At the molecular level, metabolites can provide a bridge to connect the gut microbiome with its host [46,47]. Within thousands of metabolites of biological significance, tryptophan, an essential amino acid, is metabolized into multiple microbial metabolites, such as indoles and its derivatives, which are potent bioactive metabolites that affect murine intestinal barrier integrity and immune cells [48,49]. These microbial metabolites also play an important role in inflammation, aging, neurological diseases, and environmental exposures [50,51,52,53]. Despite these findings, the metabolic reprogramming (the upregulation and downregulation of metabolites and metabolic pathways, enriched enzymes, etc.) of the gut microbiome due to MPs exposure requires further investigation.

In this pilot study, an in vitro approach was applied to expose three strains of gut bacteria, *Escherichia coli* MG1655 (*E. coli*), Nissle 1917, and *Lactobacillus rhamnosus* (*L. rhamnosus*), to MPs to investigate gut microbiome disruption and the following metabolic consequences. *E. coli* is one of the most common commensal human GI bacteria that also contributes to gut microbiome maintenance [54]. Nissle 1917 has been commonly used as a probiotic due to its ability to reduce high levels of opportunistic bacteria and return microbiome homeostasis [55,56]. Also a widely accepted probiotic, *L. rhamnosus* has been used successfully in toxicological studies on various environmental pollutants and improved gut dysmotility and neurological toxicities [57,58,59]. Our objective is to investigate how MPs modulate host gut microbiome and their metabolites using mass spectrometry (MS)-based untargeted and targeted metabolomics platforms to evaluate the metabolic alterations induced by different doses of polystyrene MPs in the bacterial culture medium. Moreover, mouse fecal microbiota was extracted and 16S rRNA sequencing was employed to examine MPs-induced modifications in taxonomic data. The present study employed a multi-omics approach to test our working hypothesis that MPs cause comprehensive changes to otherwise healthy gut microbiomes and provide insight into the mechanistic effects of MPs exposure in humans.

## 2. Materials and Methods

### 2.1. Gut Bacteria and Chemicals

Three bacterial strains were chosen for this study. *E. coli* and Nissle 1917 were generously provided by Dr. Xuan Wang’s lab at Arizona State University. Both strains were cultured in Luria Bertani (LB) medium (Difco, Detroit, MI, USA), the most commonly used growth medium for *E. coli* that promotes fast growth. *L. rhamnosus* (ATCC NO. 7469) was purchased from American Type Culture Collection (ATCC, Manassas, VA, USA), and cultured in de Man Rogosa and Sharpe (MRS) medium (MilliporeSigma, Burlington, MA, USA) that is known to contain special growth factors for *Lactobacillus* as well as a rich nutrient base.

Polystyrene MPs are one of the most abundant MPs polluted in ecosystems and aquatic environments [60,61]. As a result, polystyrene MPs (1 µm) were obtained from Phosphorex (Hopkinton, MA, USA). LC-MS grade water, methanol (MeOH), acetonitrile (ACN), isopropanol (IPA), and formic acid were obtained from Fisher Scientific (Pittsburgh, PA, USA). PBS, ammonia acetate, and ammonium hydroxide (NH_4_OH) were obtained from Sigma-Aldrich (St. Louis, MO, USA). The standard compounds corresponding to the measured metabolites were purchased from Sigma-Aldrich (Saint Louis, MO, USA) and Fisher Scientific (Pittsburgh, PA, USA).

### 2.2. Bacterial Culture and Growth Assay

All bacteria were cultured at 37 °C in an anaerobic chamber (Whitley Workstation DG250, Microbiology International, Frederick, MD, USA) under 80% N_2_, 10% H_2_, and 10% CO_2_. LB Broth and MRS Broth were equilibrated in the anaerobic environment prior to use. Bacteria were stimulated with various concentrations (0, 10, 20, 50, 100, and 500 µg/mL) of MPs in 96-well plates for 6 and 24 h, respectively, and then analyzed using Microplate Readers (BioTek Synergy H1 Microplate Reader, Winooski, VT, USA) to measure optical density (OD) at 600 nm. After 24 h of incubation at 37 °C, bacterial samples were stained for RNA-sequencing or stored at −80 °C for further analysis.

### 2.3. Fluorescence Assay

The LIVE/DEAD™ BacLight™ Bacterial Viability Kit (for microscopy, L-7007, Invitrogen, Waltham, MA, USA) was used to determine the live-dead status of the bacteria. The kit utilizes SYTO9 dye (green) to visualize live cells and propidium iodide (PI; red) to visualize the dead cells. A 1:1 mixture of SYTO9-PI reagent (3 μL) was added to 1 mL of diluted sample, mixed thoroughly, and incubated at room temperature in the dark for 15 min. Then, 5 μL of the stained bacterial suspension was trapped between a slide and an 18 mm square coverslip. Slides were observed under fluorescence microscopes, and images were captured using a KEYENCE BZ-X810 microscope (Elmwood Park, NJ, USA) with an oil-immersion objective lens at ×60 magnification. The images were processed with EZ-C1 2.20 software.

### 2.4. DNA Extraction from Mouse Fecal Samples

Fecal samples were collected from C57BL/6 mice (8-weeks old) and immediately suspended in GIFU media under anaerobic conditions. The fecal samples were homogenized with bead beating for 2 min and co-incubated with 100 µg/mL MPs for 24 h. Genomic bacterial DNA in the fecal samples was extracted and purified using E.Z.N.A.^®^ Stool DNA Kit (Omega Bio-Tek, Norcross, GA, USA) according to the manufacturer’s instructions. The concentration of DNA was determined using the Multimode Microplate Reader (BioTek, Winooski, VT, USA).

### 2.5. 16S rRNA Gene Sequencing Analysis

Extracted DNA was sent to Novogene (Sacramento, CA, USA) for Illumina MiSeq amplicon sequencing of the bacterial 16S rRNA gene using primers targeting the V3–V4 region PCR amplification with slightly modified versions of the primers 341F (5′-barcode-CCTAYGGGRBGCASCAG-3′) and 805R (5′-barcode-GGACTACNNGGGTATCTAAT-3′). Amplification and sequencing of bacterial 16S rRNA were conducted using a HiSeq-2500 sequencing system (250 bp paired-end; *n* = 4 per group). DADA2 [62] was used to collectively analyze sequences in Quantitative Insights Into Microbial Ecology (QIIME2) [63,64]. Various Python scripts were developed to analyze raw data in FASTQ format in QIIME2 including measurements of alpha-and beta-diversity and operational taxonomy unit (OTU) selection, as well as sample sequence assigning, filtering of low quality reads, format conversions, and visualizations.

### 2.6. Untargeted Metabolomics Analysis

We followed similar approaches as those in previous studies [65,66,67], and we analyzed a total of 1753 metabolites. Briefly, after collecting the medium in Eppendorf (EP) tubes, the bacteria in each well were transferred to 1.5 mL EP tubes and centrifuged at 5000× *g* for 5 min. The bacterial pellets were washed twice with ice-cold phosphate-buffered saline (PBS) and collected by centrifugation at 21,694× *g* for 5 min. To extract the aqueous metabolites from bacteria, the pellet was placed on dry ice and mixed with 1 mL of ice-cold 80% methanol (MeOH) to quench metabolism. The bacterial cell wall was disrupted by sonication on ice for 30 s (Q125 Sonicator, Qsonica, Newtown, CT, USA). The supernatant was collected after centrifugation at 14,000 rpm for 10 min, and dried completely using a Speedvac concentrator (Thermo Fisher Scientific, Waltham, MA, USA) at 30 °C.

For preparing the medium sample, 50 µL of medium was mixed with 550 µL of ice-cold MeOH containing internal standards (^13^C_3_-lactate and ^13^C_5_-^15^N-glutamic acid). The mixture was then centrifuged at 21,694× *g* for 10 min, and 450 µL of the supernatant was collected and completely dried using a vacuum drier. After adding 200 µL of acetonitrile (ACN):PBS:water (6:2:2, *v*:*v*:*v*) for reconstitution of the residues from bacterial and medium extracts, the samples were vortexed and sonicated to completely dissolve the residues and then filtered using 0.2 µm membrane filters before metabolic profiling.

A Thermo Fisher Vanquish UPLC system coupled with an Orbitrap Explorise 240 mass analyzer was utilized to perform untargeted metabolomics analysis. A pooled quality control (QC) sample was injected every 10 samples to ensure instrument consistency, with an injection volume of 1 µL for both positive and negative ionization modes. Chromatographic separation was achieved using a Waters XBridge BEH Amide column (2.1 × 50 mm, 2.5 µm, Waters Corporation, Milford, MA, USA) maintained at 40 °C. The mobile phase consisted of eluent A (0.1% formic acid in water) and eluent B (0.1% formic acid in ACN), with a flow rate of 0.3 mL/min. Gradient elution was performed as follows: 0–0.5 min, 90% of B; 0.5–6.5 min, 90–40% of B; 6.5–9.0 min, 40% of B; 9.0–9.5 min, 40–90% of B; and 9.5–15 min, 90% of B. The Orbitrap resolution was set to 120,000 with a full scan range (*m/z*) of 70–800. Full scan ddMS2 and AcquireX data acquisition with the Orbitrap resolution set at 60,000 were employed to acquire the MS/MS fragmentation pattern of detected precursor ions for metabolite identification.

Thermo Compound Discoverer 3.3 software was used for aqueous metabolomics data processing, identifying features from MS1 and MS2 spectra. MS spectra were compared against various databases, including an in-house database of approximately 300 metabolites, HMDB, METLIN database, mzCloud, Metabolika, and ChemSpider. To ensure accurate peak detection, a threshold of 100,000 was set, and the mass accuracy limit was set to 5 ppm. Only signals or peaks with a coefficient of variation (CV) less than 20% across QCs and present in more than 80% of all samples were included to enhance analysis rigor.

### 2.7. Targeted Analysis of Microbial Tryptophan Metabolism

The LC-MS/MS method employed in this study for targeted analysis of tryptophan metabolism-related microbial metabolites was developed and has been utilized in numerous published studies [41,42,43,68,69]. We measured fourteen tryptophan-related metabolites that were confirmed with the use of chemical standards. Briefly, samples were analyzed using an Agilent 1290 UPLC-6495 QQQ-MS system. 6 µL of each sample was injected onto a Waters Xbridge BEH Amide column (2.1 × 150 mm, 2.5 µm, Waters Corporation, Milford, MA, USA), with the samples in a 4 °C auto-sampler. The flow rate was set to 0.3 mL/min, and the column temperature was maintained at 40 °C. The mobile phase was composed of mobile phase A (10 mM ammonium acetate, 10 mM ammonium hydroxide in 95% water/5% ACN) and B (10 mM ammonium acetate, 10 mM ammonium hydroxide in 95% ACN/5% water). Gradient elution was performed as follows: 0–1 min, 90% of B; 1–11 min, 90–40% of B; 11–15 min, 40% of B; and then B% returned to 90% in 0.5 min. The mass spectrometer was equipped with an electrospray ionization source, and data acquisition was performed in multiple-reaction-monitoring (MRM) mode under positive ionization mode. The data acquisition was controlled by Agilent MassHunter Workstation software, and the data integration and extraction were performed using Agilent MS Quantitative Analysis software.

### 2.8. Statistical Analysis

Univariate and multivariate statistical analysis, as well as volcano plots and pathway and enzyme enrichment analyses were performed using the MetaboAnalyst 6.0 software [70]. Taxonomic data, including α diversity (Chao1 index) metrics, were analyzed using QIIME2. A threshold α-level of 0.05 was used to define statistical significance, and false discovery rate (FDR) was applied to all analyses unless otherwise stated.

## 3. Results

### 3.1. MPs Reduce Growth of Selected Probiotics

**Figure 1:**
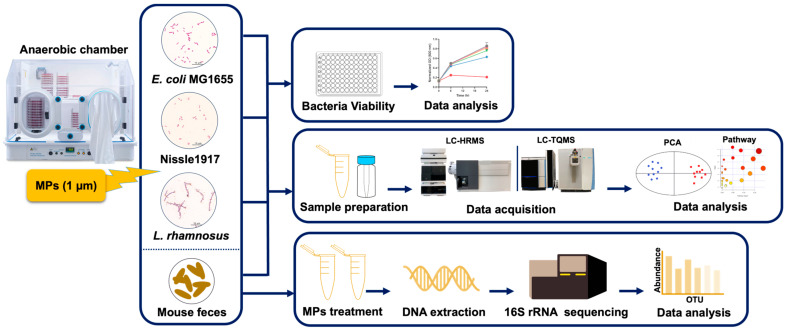
The overall scheme of the research processes.

**Figure 2:**
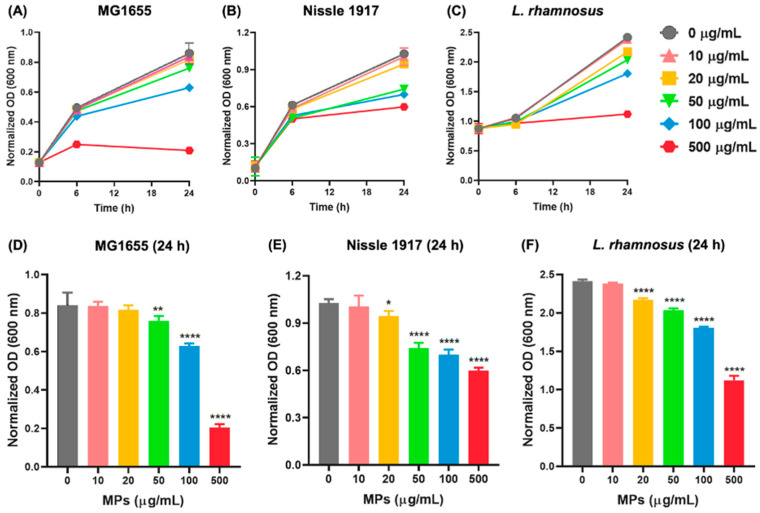
MPs induce decrease of bacterial viabilities. (**A**,**D**) *E. coli*, (**B**,**E**) Nissle 1917, and (**C**,**F**) *L. rhamnosus* growth in response to MPs treatment (0, 10, 20, 50, 100, 500 µg/mL) for 24 h. Cell density was measured from optical density (600 nm). Data are presented as mean ± SD (*n* = 6). * *p* < 0.05, ** *p* < 0.01 and **** *p* < 0.0001 versus 0 µg/mL.

Figure 1 provides a schematic overview of the experiments in this study. First, we examined the impacts of MPs on bacterial growth at the strain level. Figure 2 shows the gut bacteria cell growth within 24 h after different concentrations of MPs exposure for *E. coli*, Nissle 1917, and *L. rhamnosus*. It was found cell densities (indicated by OD values) decreased in a dose-dependent manner; for example, *E. coli* had significantly lower OD values if exposed to ≥50 µg/mL of MPs for 24 h (Figure 2A,D), while Nissle 1917 and *L. rhamnosus* OD values significantly decreased within the concentration range of 20–500 µg/mL (Figure 2B,C,E,F). Therefore, concentrations of 50 and 100 µg/mL MPs were selected for metabolomics analysis for *E. coli*, and concentrations of 20 and 100 µg/mL MPs were used for Nissle 1917 and *L. rhamnosus*.

**Figure 3:**
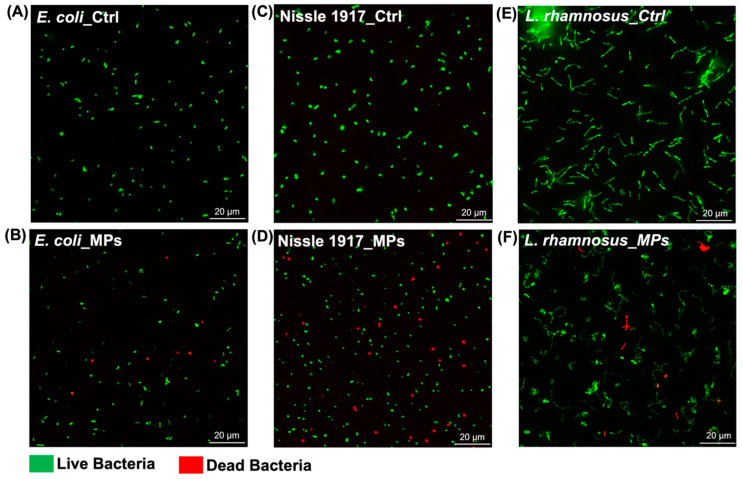
Fluorescence staining of (**A**,**B**) *E. coli*, (**C**,**D**) Nissle 1917, and (**E**,**F**) *L. rhamnosus* which were incubated with broth with or without MPs (100 µg/mL) for 24 h, respectively. Scale bars, 20 µm. Live bacteria with intact cell membranes emit green fluorescence, whereas dead bacteria with damaged membranes give red fluorescence.

Immunofluorescence microscopy was conducted using the BacLight LIVE/DEAD staining kit. All bacterial strains were cultured with or without 100 µg/mL MPs in the medium, respectively. After 24 h of exposure, bacteria were stained with SYTO9 and PI dyes and observed using a KEYENCE microscope. As shown in Figure 3A–C, all bacterial cells in the Control group (no MPs) predominantly exhibited green fluorescence, appearing as separate spots or stripes. In contrast, exposure to MPs led to a significant increase of red fluorescence in *E. coli*, Nissle 1917, and *L. rhamnosus* (Figure 3D–F), suggesting higher death rates compared to bacteria without MPs exposure.

### 3.2. MPs Exposure Caused Metabolic Programming in a Wide Metabolome

Untargeted metabolomics was performed to characterize the responsive metabolites of the three different model bacteria exposed to MPs. Figure S1 shows the volcano plots of the bacterial and medium metabolites created using MetaboAnalyst, comparing the metabolomic profiles of control groups versus MPs exposure groups (100 µg/mL) in *E. coli*, Nissle 1917, and *L. rhamnosus*. Tables S1–S6 listed the corresponding significant metabolites and their fold changes comparing control and MPs exposed groups. These univariate data analysis results showed that MPs exposure induced significant changes in a wide metabolome.

**Figure 4:**
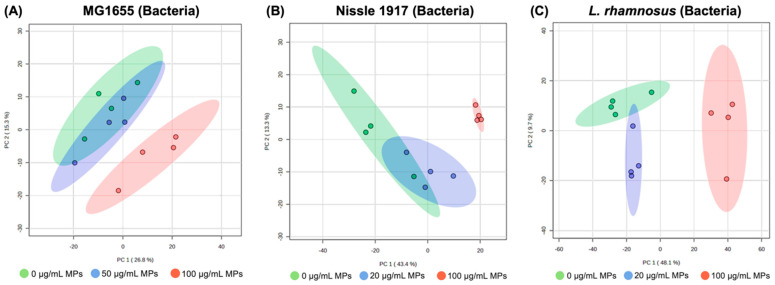
PCA score plots of the untargeted metabolomics data from three different gut bacteria. (**A**) *E. coli*, (**B**) Nissle1917, and (**C**) *L. rhamnosus.* Green dots: control group; blue dots: 50 µg/mL (*E. coli*) or 20 µg/mL (Nissle1917 and *L. rhamnosus*) MPs exposure group; red dots: 100 µg/mL MPs exposure group.

In *E. coli*, following the 24 h exposure, the principal component analyses (PCA) score plot showed that the control group is well differentiated from the 100 µg/mL MPs exposure group with the 50 µg/mL MPs exposure group trending in the same direction (Figure 4A). As shown in Figure 4B,C, MPs-exposed Nissle 1917 and *L. rhamnosus* groups displayed pronounced separation between the control groups. The 20 µg/mL MPs exposure groups were not completely differentiated, but the 100 µg/mL MPs exposure groups demonstrated clear group differences. This observation indicated significant metabolic disturbances and clear alterations in metabolism induced by MPs.

### 3.3. Pathway and Enzyme Enrichment Analysis on Individual Gut Microbes

**Figure 5:**
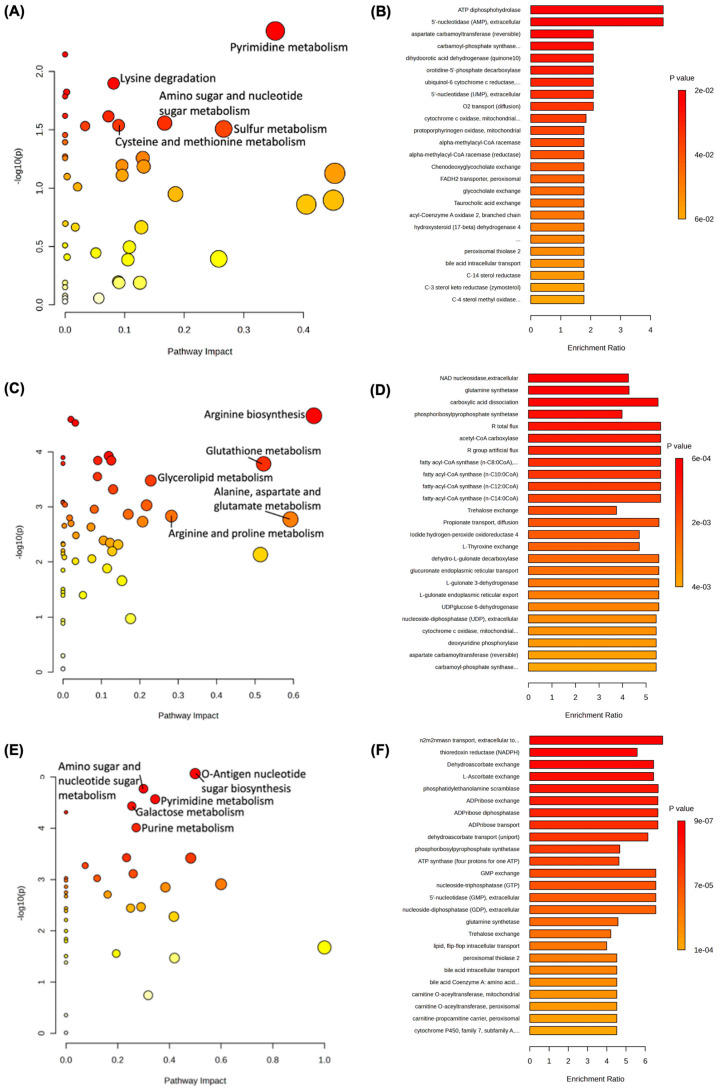
Metabolic pathway and enzyme enrichment analyses of three different gut bacteria after 100 µg/mL MPs exposure. (**A**,**B**) *E. coli*, (**C**,**D**) Nissle 1917, (**E**,**F**) *L. rhamnosus*. The metabolic pathways are represented as circles according to their scores of enrichment (vertical axis, shade of red) and topology (pathway impact, horizontal axis, circle diameter) analysis using MetaboAnalyst 6.0. Estimated enriched enzymes are depicted by *p*-values and enrichment ratios.

Pathway analysis of MPs-exposed *E. coli* bacteria compared to controls revealed significant alterations (*p* < 0.05) in shared pathways contributing to (i) pyrimidine metabolism, (ii) histidine metabolism, (iii) lysine degradation, (iv) cysteine and methionine metabolism, (v) sulfur metabolism, and (vi) amino sugar and nucleotide sugar metabolism (Figure 5A and Table 1). However, enzyme enrichment analyses for estimated activity were conducted and found no significantly enriched intracellular enzymes (Figure 5B).

In the 100 µg/mL MPs-exposed Nissle 1917 bacteria group, (i) glycerolipid metabolism, (ii) alanine, aspartate and glutamate metabolism, (iii) arginine and proline metabolism, (iv) arginine biosynthesis, and (v) glutathione metabolism were similarly disturbed (Figure 5C). Enzyme enrichment analyses were also conducted and found several intracellular enriched enzymes, including (i) nicotinamide adenine dinucleotide (NAD) nucleosidase, (ii) glutamine synthetase, (iii) acetyl CoA carboxylase, and (iv) fatty acyl-CoA synthase (Figure 5D).

The MPs-exposed *L. rhamnosus* bacteria group found significant changes to nucleotide and sugar metabolism, including (i) amino sugar and nucleotide sugar metabolism, (ii) O-Antigen nucleotide sugar metabolism, (iii) galactose metabolism, (iv) pyrimidine metabolism, and (v) purine metabolism (Figure 5E). While the MPs-exposed Nissle 1917 group appeared to affect more amino acid metabolites, both MPs-exposed *E. coli* and *L. rhamnosus* bacteria shared common impacts in amino sugar and nucleotide sugar metabolism and pyrimidine metabolism. In addition, enzyme enrichment analyses were conducted and uncovered several significant intracellular enzymes related to (i) NADPH, (ii) L-ascorbate exchange, (iii) ADP-ribose, (iv) ATP synthase, (v) GMP (guanosine monophosphate), GDP, and GTP, and (vi) glutamine synthetase (Figure 5F).

### 3.4. Mouse Fecal Microbiota Exposed to MPs

In order to further explore the potential effects of MPs exposure on various gut microbiota, we obtained C57BL/6 mouse fecal samples. We extracted gut microbiota from the fecal samples and cultured them with 100 μg/mL MPs for 24 h. We first analyzed the alterations of bacterial components in the samples using 16S rRNA sequencing (*n*  =  4 per group). In Figure S2, QIIME2 was used to calculate the α diversity (Chao1 index) metrics for all the rarefied OTU tables. The α diversity quantifications demonstrated no marked differences between control and MPs exposure groups.

**Figure 6:**
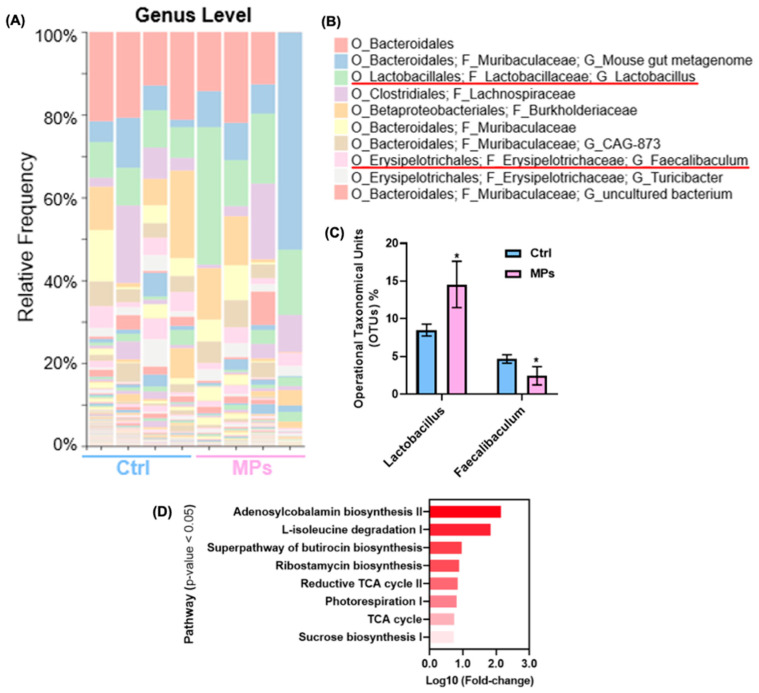
The effects of MPs exposure to the gut microbiome extracted from C57BL/6 mouse fecal samples. (**A**) The relative frequency of gut microbes at the genus level from the control and MPs groups, (**B**) ten most abundant taxa identified by QIIME2 (red line indicates analyzed taxa), (**C**) operational taxonomical units of *Lactobacillales* and *Erysipelotrichales* in Control vs. MPs groups, * *p* < 0.05, and (**D**) functional analysis results shown as metabolic pathways vs. log-transformed fold changes.

Figure 6A shows the relative frequency of gut microbes at the genus level in the control and MPs groups. A total of 25 phyla were identified by QIIME2, with the ten most abundant taxa shown in Figure 6B. In addition, 16S rRNA sequencing results showed an upregulation (*p* < 0.05) of *Lactobacillales* and a reduction (*p* < 0.05) in *Erysipelotrichales* due to MPs exposure (Figure 6C). In Figure 6D, functional analysis (metabolic pathway vs. log-transformed fold change) showed alterations in energy producing pathways, such as (i) reductive TCA (tricarboxylic acid cycle) cycle II, (ii) photorespiration, (iii) TCA cycle, and (iv) sucrose biosynthesis I. Additional impacted pathways included (v) adenosylcobalamin biosynthesis II, (vi) L-isoleucine degradation I, (vii) superpathway of butirocin biosynthesis, and (viii) ribostamycin biosynthesis.

### 3.5. Metabolic Reprogramming in Mouse Fecal Microbiome Exposed to MPs

Both untargeted and targeted metabolomics were performed on the gut bacteria (extracted from mouse feces) and medium samples. Figure S3 shows the volcano plots of the bacterial and medium metabolites, comparing the metabolomic profiles of control groups versus MPs exposure groups (100 µg/mL for 24 h). Tables S7 and S8 listed the corresponding significant metabolites and their fold changes comparing control and MPs exposed groups in bacteria and medium, respectively. It is obvious that metabolic profiles were significantly altered due to MPs exposure.

**Figure 7:**
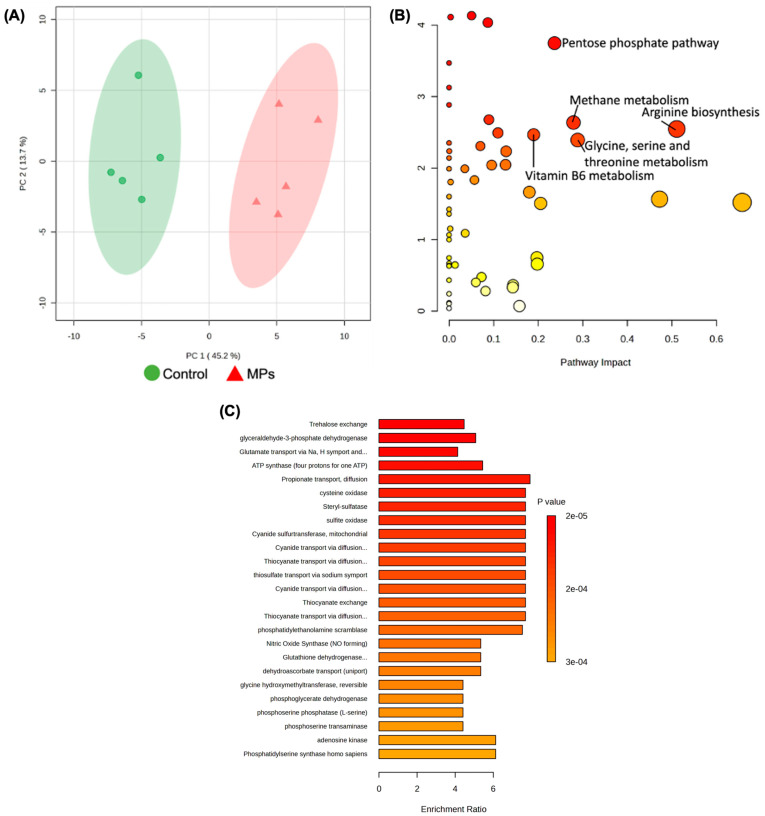
(**A**) PCA score plot of the untargeted metabolomics data from the mouse fecal microbiota samples with and without MPs exposure (100 μg/mL MPs for 24 h), (**B**) metabolic pathway analysis, and (**C**) enrichment analyses of intracellular enzymes using MetaboAnalyst 6.0. The metabolic pathways are represented as circles according to their scores of enrichment (vertical axis, shade of red) and topology (pathway impact, horizontal axis, circle diameter). Enzyme enrichment is plotted as enrichment ratio, and more significant *p*-values are denoted by darker shade of red.

As shown in Figure 7A, the untargeted metabolic profiles of the control and MPs groups are well separated in the PCA score plot, with 45.2% and 13.7% variation explained by PC1 and PC2, respectively. Pathway analysis of the MPs-exposed mouse gut microbiota samples compared to control samples revealed significant alterations (*p* < 0.05) in shared pathways contributing to (i) arginine biosynthesis, (ii) pentose phosphate pathway, (iii) methane metabolism, (iv) glycine, serine, and threonine metabolism, and (v) vitamin B6 metabolism (Figure 7B and Table 2). Enzyme enrichment analyses for estimated activity were conducted and found significant activity with intracellular enzymes related to (i) glutamate transport, (ii) ATP synthetase, (iii) propionate transport (iv) sulfur metabolism, and (v) nitric oxide synthase (Figure 7C).

**Figure 8:**
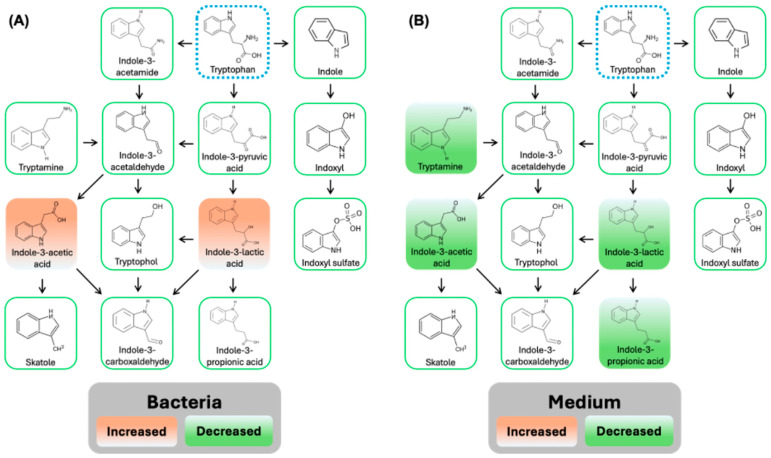
The impacts of MPs exposure on microbial tryptophan metabolism in gut microbiota extracted from C57BL/6 mouse fecal samples. (**A**) mouse fecal microbiota, and (**B**) medium samples. Orange: significantly increased (*p* < 0.05); green: significantly decreased (*p* < 0.05).

Microbial tryptophan metabolism plays an important role in gut intestinal barrier integrity, inflammation, aging, and neurological diseases [49,50,51,52,53]. Since tryptophan can be metabolized by gut microbiota into indole and its derivatives, Figure 8 shows the changes of metabolites in microbial tryptophan metabolism impacted by MPs exposure (100 μg/mL MPs for 24 h). In the mouse fecal microbiome samples, indole-3-acetic acid and indole-3-lactic acid are both significantly decreased (*p* < 0.05) by MPs exposure. In the medium samples, tryptamine, indole-3-acetic acid, indole-3-lactic acid, and indole-3-propionic acid are significantly downregulated (*p* < 0.05) by MPs exposure.

## 4. Discussion

In this study, we employed a multi-omics approach to test our working hypothesis that microplastics (MPs) cause comprehensive changes to otherwise healthy gut microbiome and provide insights into the mechanistic effects of MPs exposure in humans. Immunofluorescence microscopy demonstrated reductions in bacterial growth after 24-h exposure to MPs at the strain level. In addition, untargeted and targeted metabolomics platforms found significant metabolic alterations induced by various doses of polystyrene MPs in the examined bacterial strains. Moreover, extracted mouse fecal microbiota and 16S rRNA sequencing revealed significant MPs-induced modifications in taxonomic data.

Due to the ubiquity of plastics in our society and its widespread pollution, humans are exposed to MPs daily. In fact, recent reports leveraging novel imaging techniques have been used to identify greater than a thousand MPs in common single-use water bottles [71]. Regulatory agencies are currently working to establish guidelines and standards for acceptable levels of MPs in food and water as it is the primary method of entering the human body [72]. Once ingested, MPs may translocate through the gastrointestinal (GI) tract, enter the bloodstream, and reach other organs like the liver and brain [73]. MPs can adsorb and accumulate various chemicals from the environment, including pollutants and additives used in the manufacturing process [74]. There are rising concerns that these chemicals may leach from MPs once inside the human body, leading to additional exposure to harmful substances [75]. Previous studies suggest that MPs may induce inflammatory responses in the body, which can result in chronic inflammation and lead to various health problems, including cardiovascular disease (CVD), T2D, and other conditions [76]. It is postulated that MPs promote platelet aggregation, oxidative stress, and cell senescence [76]. MPs may accumulate within vascular lesions and atherosclerotic plaques resulting in increased CVD risk [76].

Human GI microbiota, also known as the gut microbiome, is a range of microorganisms that reside in the GI tract and aid digestion [32]. They play important roles in human metabolism, nutrition, physiology, and immune function [22,77]. The presence of MPs in the GI tract may lead to harmful interactions within the gut microbiome, potentially influencing its composition and function [78]. This disruption in the gut microbiota could have downstream negative influences on human health [32,33]. A potential reduction of essential microbial groups needed for gut balance and an increase of pro-inflammatory and disease-related bacterial groups would result in altered intestinal homeostasis [34,35].

In this study, *E. coli* was selected because it is known for its contributions to gut microbiome maintenance and is considered as one of the most common commensal human GI bacteria [54]. As a common probiotic, Nissle 1917 has shown the ability to reduce high levels of opportunistic bacteria for microbiome homeostasis [55,56]. Moreover, it can help facilitate fibrous matrices that promote gut epithelial integrity and reduce pathogen translocation [79]. *L. rhamnosus* is also a commonly used probiotic for the prevention and treatment of GI infections and immune response activation for alleviating allergic symptoms [80]. As our results demonstrate, the exposure to MPs diminished the survival rates of all three bacterial strains in a dose-dependent manner. Metabolomics analyses of the three bacteria at varied MPs exposure also confirmed metabolic differences between the control and MPs groups, increasing in significance as the dosage increased.

Metabolic pathway analyses at the strain level of the three bacteria mainly highlighted alterations to amino acid, nucleotide, and sugar metabolism, which included pyrimidine metabolism, amino sugar, and nucleotide sugar metabolism in both *E. coli* and *L. rhamnosus*. Pyrimidine metabolism in bacteria is a highly complex and regulated process that involves essential components of nucleic acids, which play a critical role in various cellular processes, including DNA replication, transcription, and translation [81]. The Nissle 1917 impacted pathways were largely single chain amino acid metabolism, including arginine biosynthesis, arginine and proline metabolism, and alanine, aspartate, and glutamate metabolism. The metabolism of arginine and other amino acids can be influenced by various interactions between the gut microbiota and other body organs like the liver [82,83,84]. As a semi-essential amino acid, intraluminal arginine levels may not adequately meet metabolic demands. Disruptions in amino acid metabolism have been linked to a variety of immune-mediated or infectious diseases in the gut [85,86]. Interestingly, the sulfur metabolic pathway in *E. coli* was also significantly altered after exposure, and each of the bacterial strains had many significantly affected sulfur derivatives, such as lauryl sulfate, dodecyl sulfate, sulfamic acid, sulfurous acid, and sulfuric acid [87]. As a crucial component of bacterial amino acids and cellular function, sulfur is essential for microbial homeostasis, growth, and survival [88].

Moreover, the altered metabolism of nucleotide sugars aligns with our results in the estimated activity of enriched enzymes. Both strains of *E. coli* and Nissle 1917 had significantly enriched enzymes linked to UMP (uridine monophosphate) and UDP metabolism, while *L. rhamnosus* demonstrated shifts in GMP (guanosine monophosphate), GDP, and GTP metabolism. All five compounds and their intermediaries are important components in amino sugar and nucleotide sugar metabolism [89]. These metabolic processes are essential in regulating protein function and the formation of polysaccharides and are linked with developing several conditions like T2D, neurodegenerative disorders, and infectious disease [90,91]. In addition, multiple carnitine derivatives (DL-carnitine, 9-hexadecenoyl carnitine and O-(17-carboxyheptadecanoyl) carnitine), long chain fatty acids (juniperic acid and 3-oxopalmitic acid), and intermediates of glycolysis and the TCA cycle (phosphoenolpyruvic acid and fumaric acid) were among the most significantly altered metabolites of all three strains, which may signify differences in beta-oxidation and energy production [92]. These effects of MPs exposure on cellular respiration processes potentially indicate a mechanism for the reduced bacterial growth.

MPs-treated mouse fecal microbiota samples were analyzed to investigate induced genomic and metabolomic alterations to the gut microbial composition and metabolome. While no significant α diversity was observed after 100 μg/mL MPs exposure for 24 h, two bacteria, *Lactobacillales* and *Erysipelotrichales*, significantly changed at the genus level. The PCA score plot of control and MPs exposed groups demonstrate significant metabolic differences occurring after the treatment. Metabolic pathway analysis of the fecal microbiome samples revealed alterations to arginine biosynthesis and alanine, aspartate, and glutamate metabolism. Interestingly, D-amino acid metabolism is significantly different between the groups. Bacterial cells produce a large quantity of D-amino acids in the gut microbiome and the difference may be due to the reduced bacterial growth in the MPs exposed group [93]. Additionally, altered methane metabolism and the reduced prevalence of enriched sulfur-related enzymes were observed after MPs treatment. Methane and sulfur derivatives are primarily produced through the anaerobic microbial breakdown of organic matter and are normal byproducts of the gut microbial community [88]. Similar to the bacterial strain findings, energy producing pathways like glycolysis, gluconeogenesis, and the pentose phosphate pathway were significantly altered after MPs exposure. The enzyme enrichment analyses also uncovered shifts in significantly enriched enzymes like intracellular ATP synthetase.

A targeted examination of microbial tryptophan metabolism was employed to investigate the effects of MPs exposure on the gut microbiome. Tryptophan and its derivatives have been shown to be integral in gut intestinal barrier integrity and associated with conditions like inflammation, aging, and neurological diseases via the gut-brain-axis [29,49,50,51,52,53]. Tryptophan can be metabolized by the gut microbiota into indole and its derivatives, such as indole-3-lactic acid (ILA), indole-3-acetic acid (IAA), and indole-3-propionic acid (IPA). Our targeted analyses identified significantly disrupted microbial tryptophan metabolism in both the fecal microbiota and medium samples. Specifically, ILA and IAA were significantly decreased in the mouse fecal microbiome samples by MPs exposure, and tryptamine, ILA, IAA, and IPA were significantly downregulated in the medium samples. The conversion of tryptophan to indole requires tryptophanase (TnaA) activation, which occurs in nearly all bacterial species, including *E. coli*, *Bacteroides* sp., and *Clostridium* sp. [50,94]. The production of the intermediate ILA requires the aromatic amino acid aminotransferase and ILA dehydrogenase–dependent pathway in *Lactobacillus* and *Bifidobacterium* [95,96]. Likewise, the conversion of ILA to IPA requires the presence of phenyllactate dehydratase gene cluster (fldAIBC) in *Clostridum* and *Peptostreptococcus* [97]. The decarboxylation of tryptophan by tryptophan decarboxylases (TrpDs) in commensal bacteria can also produce tryptamine [50]. Moreover, the conversion of tryptophan to tryptamine in a TrpD-dependent manner has been identified in *Clostridium*, *Ruminococcus*, *Blautia*, and *Lactobacillus* [50].

There are a few limitations of this early pilot study. The bacterial strains used in this study were from the phyla *Proteobacteria* and *Firmicutes*, while bacteria from *Bacteroidetes*, *Actinobacteria*, *Fusobacteria*, and *Verrucomicrobia* were not included. We acknowledge that using three commensal GI bacteria for MPs testing is not representative of the entire gut microbiome. In addition, the size, concentrations, and types of MPs used in this study do not represent all forms of MPs and nanoplastics (NPs) unique to our society and environment. Many other forms of MPs like polyethylene, polypropylene, polyamide, polyester, and acrylic plastics warrant future study. Moreover, gut microbiota was extracted from mouse fecal samples and exposed to MPs. We recognize that cultured bacterial environments are different than the gut microbiome environment and future in vivo studies are necessary to further confirm our findings.

## 5. Conclusions

The results of this study revealed that MPs exposure caused a significant reduction of bacterial viabilities for all selected bacterial strains in a dose-dependent manner. In addition, metabolomics showed that there were significant alterations in multiple metabolic pathways in the gut bacteria due to MPs exposure, especially in pyrimidine metabolism, arginine biosynthesis, nucleotide sugar, and tryptophan metabolisms, as well as energy producing pathways. PCA score plots further highlighted group differences in MPs exposure in a dose-dependent manner. Moreover, gut microbiota isolated from mice fecal samples and 16S rRNA sequencing displayed alterations in taxonomic data after MPs exposure. In particular, *Lactobacillales* and *Erysipelotrichales* were significantly changed at the genus level. Targeted analysis also showed that MPs exposure had a significant effect on microbial tryptophan metabolism. In summary, this study demonstrated that MPs exposure may cause comprehensive changes to the gut microbiota at multi-omics levels, which could provide insights to understanding the mechanistic effects of MPs exposure in humans. Future directions may include in vivo experiments to examine health effects after MPs exposure.

## Figures and Tables

**Table 1:** Summary of pathways impacted by MPs in three different gut bacteria.

	Pathway	*p* Value	−log10(*p*)	Impact
*E. coli*	Pyrimidine metabolism	**	2.3466	0.3527
	Sulfur metabolism	*	1.5078	0.2661
	Amino sugar and nucleotide sugar metabolism	*	1.5570	0.1671
	Lysine degradation	*	1.8973	0.0815
	Cysteine and methionine metabolism	*	1.5377	0.0897
Nissle 1917	Arginine biosynthesis	****	4.6557	0.6533
	Glutathione metabolism	***	3.7819	0.5224
	Alanine, aspartate and glutamate metabolism	**	2.7727	0.5923
	Glycerolipid metabolism	***	3.4779	0.2286
	Arginine and proline metabolism	**	2.8326	0.2823
*L. rhamnosus*	O-Antigen nucleotide sugar biosynthesis	****	5.0656	0.5000
	Amino sugar and nucleotide sugar metabolism	****	4.7709	0.2998
	Pyrimidine metabolism	****	4.5666	0.3450
	Galactose metabolism	****	4.4337	0.2545
	Purine metabolism	****	4.0119	0.2715

Compared to control group: **** *p* < 0.0001, *** *p* < 0.001, ** *p* < 0.01, * *p* < 0.05.

**Table 2:** Summary of pathways impacted by MPs in mouse gut bacteria.

	Pathway	*p* Value	−log10(*p*)	Impact
Bacteria	Arginine biosynthesis	**	2.5472	0.5110
	Pentose phosphate pathway	***	3.7471	0.2367
	Methane metabolism	**	2.6371	0.2792
	Glycine, serine and threonine metabolism	**	2.3922	0.2886
	Vitamin B6 metabolism	**	2.467	0.1898
	Glutathione metabolism	*	1.5655	0.4724
	Alanine, aspartate and glutamate metabolism	*	1.5206	0.6577
	D-Amino acid metabolism	****	4.0348	0.0870
	Glycolysis/Gluconeogenesis	*	1.6635	0.1801
	Cysteine and methionine metabolism	*	1.5061	0.2054

Compared to control group: **** *p* < 0.0001, *** *p* < 0.001, ** *p* < 0.01, * *p* < 0.05.

## Data Availability

The original contributions presented in this study are included in this article; further inquiries can be directed to the corresponding author.

## Supplementary Materials

The following supporting information can be downloaded at: https://www.mdpi.com/article/10.3390/biomedicines13020446/s1, Figure S1: Volcano plot analyses comparing control groups vs. MPs exposure groups (100 µg/mL); Figure S2: Bacterial alpha diversity of Control and MPs groups using the Chao 1 index in QIIME2; Figure S3: Volcano plot analyses comparing control groups vs. MPs exposure groups (100 µg/mL for 24 h); Table S1: Significant metabolites and fold change analysis comparing control groups vs. MPs exposure groups (100 µg/mL): MG1655 bacteria; Table S2: Significant metabolites and fold change analysis comparing control groups vs. MPs exposure groups (100 µg/mL): MG1655 medium; Table S3: Top 30 Significant metabolites and fold change analysis comparing control groups vs. MPs exposure groups (100 µg/mL): Nissle1917 bacteria; Table S4: Top 30 Significant metabolites and fold change analysis comparing control groups vs. MPs exposure groups (100 µg/mL): Nissle1917 medium; Table S5: Top 30 Significant metabolites and fold change analysis comparing control groups vs. MPs exposure groups (100 µg/mL): *L. rhamnosus* bacteria; Table S6: Significant metabolites and fold change analysis comparing control groups vs. MPs exposure groups (100 µg/mL): *L. rhamnosus* medium; Table S7: Top 30 Significant metabolites and fold change analysis comparing control groups vs. MPs exposure groups (100 µg/mL): gut bacteria extracted from C57BL/6 mouse feces; Table S8: Significant metabolites and fold change analysis comparing control groups vs. MPs exposure groups (100 µg/mL): medium samples for the gut bacteria extracted from C57BL/6 mouse feces.
