# Supplementary material for: Metabolic Reprogramming in Gut Microbiota Exposed to Polystyrene Microplastics
Source: Biomedicines. 2025 Feb 12;13(2):446. doi: 10.3390/biomedicines13020446 (PMC11853289; doi:10.3390/biomedicines13020446)
Supplement: Supplementary file 1 [file biomedicines-13-00446-s001.zip › biomedicines-3417605-supplementary.pdf]

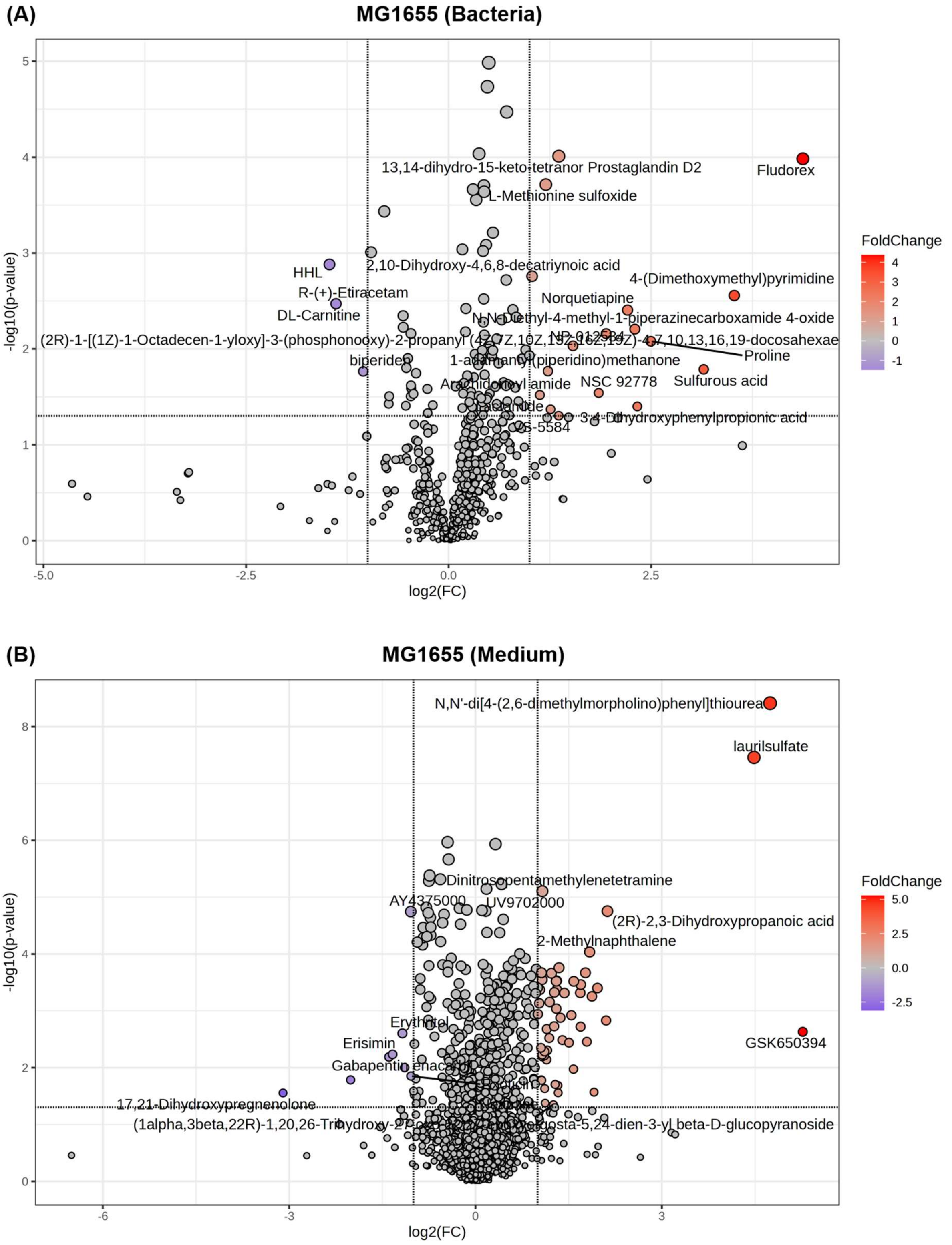
(A)
MG1655 (Bacteria)
-log10(p-value)
log2(FC)
FoldChange
13,14-dihydro-15-keto-tetranor Prostaglandin D2
Fludorex
L-Methionine sulfoxide
HHL
2,10-Dihydroxy-4,6,8-decatriynoic acid
4-(Dimethoxymethyl)pyrimidine
R-(+)-Etiracetam
Norquetiapine
DL-Carnitine
Proline
Sulfurous acid
NSC 92778
3,4-Dihydroxyphenylpropionic acid
(B)
MG1655 (Medium)
-log10(p-value)
log2(FC)
FoldChange
N,N'-di[4-(2,6-dimethylmorpholino)phenyl]thiourea
laurilsulfate
Dinitrosopentamethylenetetramine
UV9702000
(2R)-2,3-Dihydroxypropanoic acid
2-Methylnaphthalene
Erythritol
Erisimin
GSK650394
17,21-Dihydroxypregnenolone

(C)

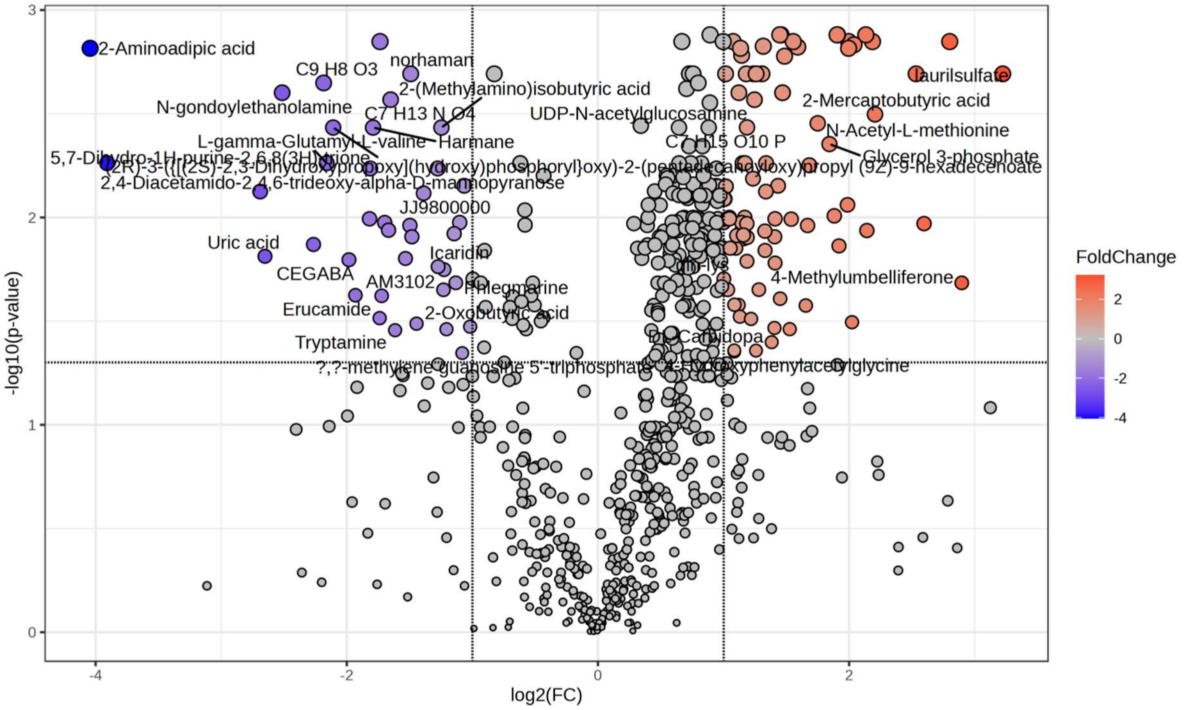
Nissle 1917 (Bacteria)
2-Aminoadipic acid
C9 H8 O3
norharman
N-gondoylethanolamine
2-(Methylamino)isobutyric acid
C7 H13 N O4
UDP-N-acetylglucosamine
L-gamma-Glutamyl-L-valine
Harmane
C7 H15 O10 P
5,7-Dihydro-1H-purine-2,6,8(3H)-trione
2,4-Diacetamido-2,4,6-trideoxy-alpha-D-mannopyranose
JJ9800000
Uric acid
Icaridin
CEGABA
AM3102
Phlegmarine
Erucamide
2-Oxobutyric acid
Tryptamine
Carbidopa
laurilsulfate
2-Mercaptobutyric acid
N-Acetyl-L-methionine
Glycerol 3-phosphate
4-Methylumbelliferone
FoldChange
log2(FC)
-log10(p-value)

(D)

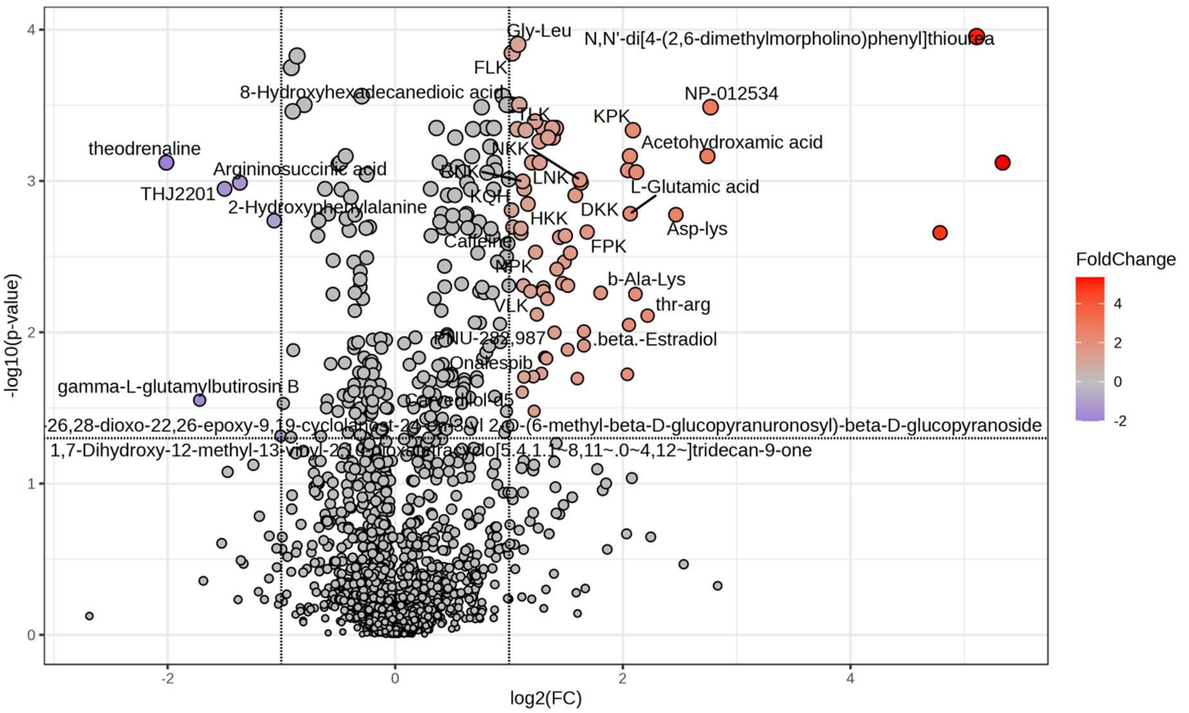
Nissle 1917 (Medium)
Gly-Leu
N,N'-di[4-(2,6-dimethylmorpholino)phenyl]thiourea
FLK
8-Hydroxyhexadecanedioic acid
NP-012534
KPK
theodrenaline
Acetohydroxamic acid
NKK
Argininosuccinic acid
LNK
THJ2201
L-Glutamic acid
2-Hydroxyphenylalanine
DKK
HKK
Asp-lys
Caffeine
FPK
b-Ala-Lys
VLK
thr-arg
.beta.-Estradiol
Onalespib
gamma-L-glutamylbutirosin B
1,7-Dihydroxy-12-methyl-13-vinyl-2,10-dioxatetracyclo[5.4.1.1~8,11~.0~4,12~]tridecan-9-one
FoldChange
log2(FC)
-log10(p-value)

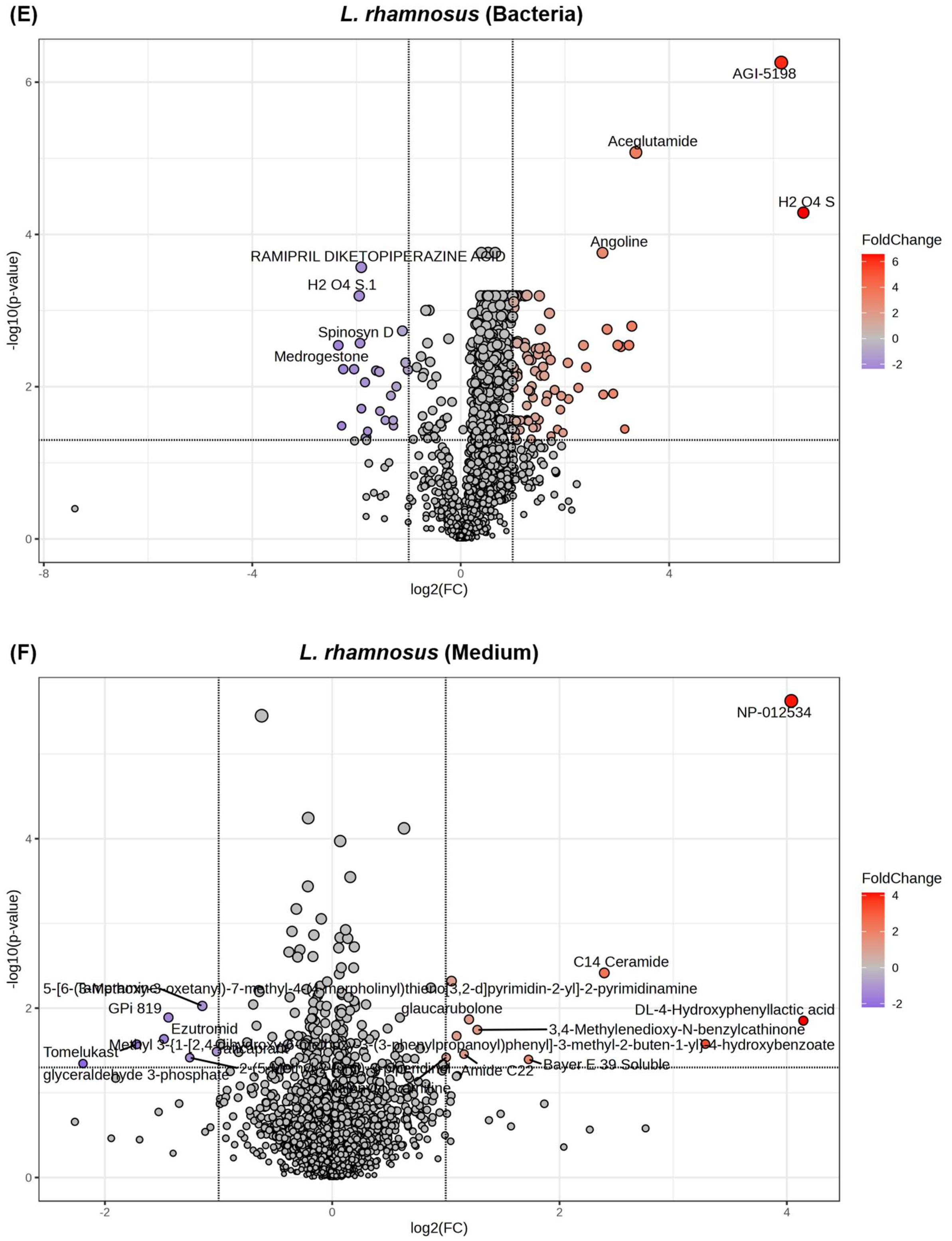

Figure S1. Volcano plot analyses comparing control groups vs MPs exposure groups (100 μg/mL). (**A**) *E. coli* MG1655 bacteria; (**B**) *E. coli* MG1655 medium; (**C**) Nissle 1917 bacteria; (**D**) Nissle 1917 medium; (**E**) *L. rhamnosus* bacteria; (**F**) *L. rhamnosus* medium.

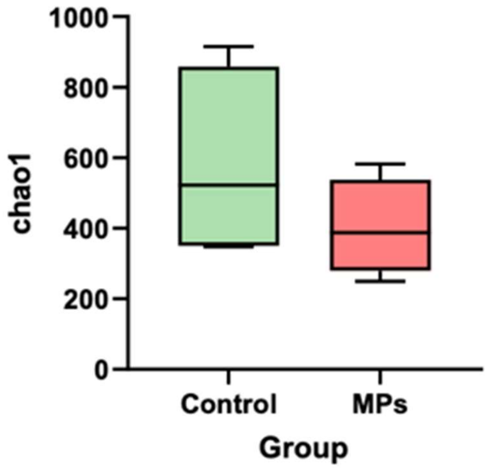

Figure S2. Bacterial alpha diversity of Control and MPs groups using the Chao 1 index in QIIME2. Gut microbiota was extracted from C57BL/6 mouse fecal samples and then cultured with 100 μg/mL MPs for 24 hours.

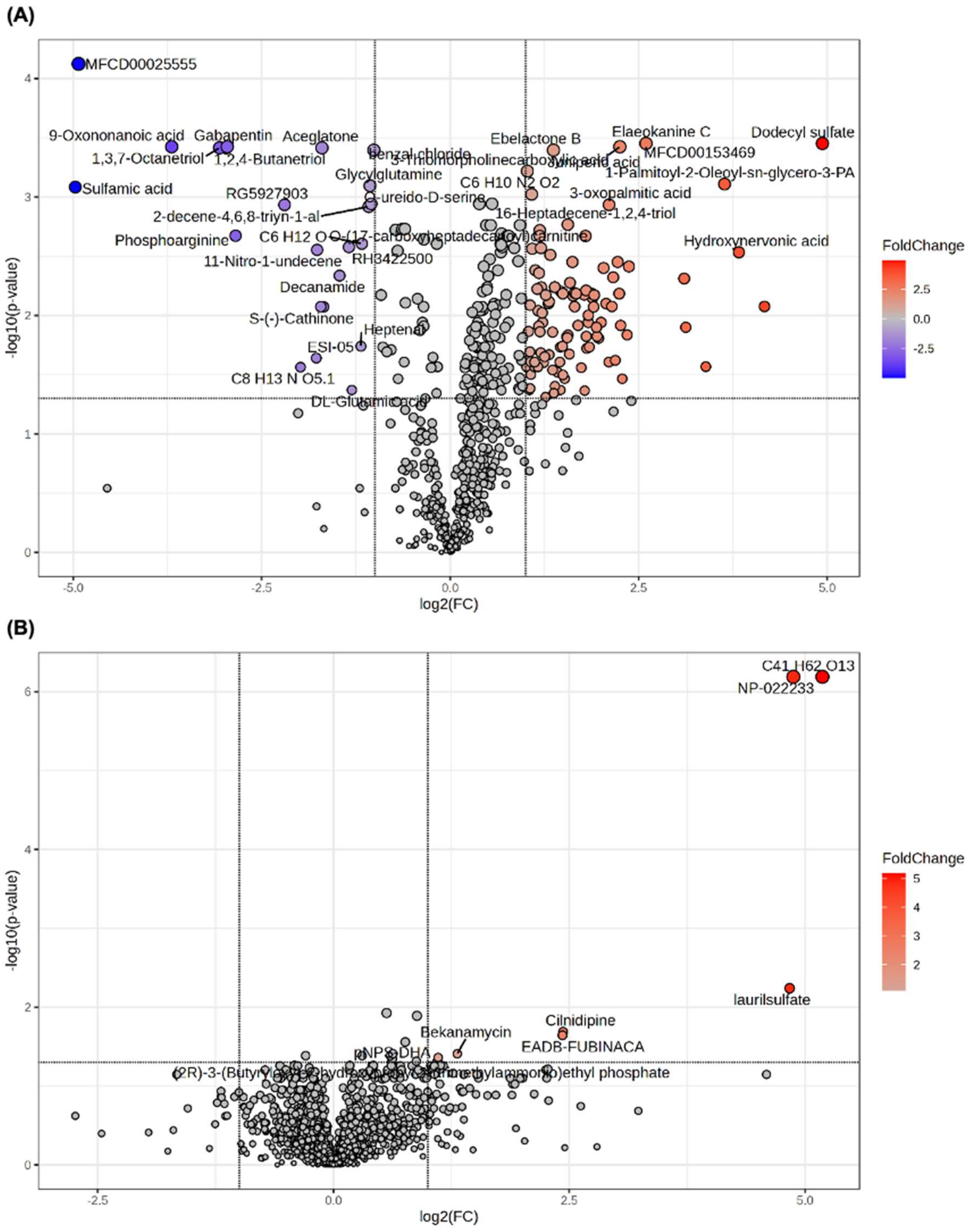

Figure S3. Volcano plot analyses comparing control groups vs MPs exposure groups (100 μg/mL for 24 hours). (**A**) gut bacteria extracted from C57BL/6 mouse feces; (**B**) medium samples.

Table S1. Significant metabolites and fold change analysis comparing control groups vs MPs exposure groups (100 μg/mL): MG1655 bacteria.

	Metabolites	MPs/Control (log2 FC)	*p*. value
1	13,14-dihydro-15-keto-tetranor PED2	↑****	9.75E-05
2	Fludorex	↑***	0.000104
3	L-Methionine sulfoxide	↑***	0.000193
4	HHL	↓**	0.001315
5	2,10-Dihydroxy-4,6,8-decatriynoic acid	↑**	0.001746
6	4-(Dimethoxymethyl)pyrimidine	↑**	0.002777
7	DL-Carnitine	↓**	0.003383
8	R-(+)-Etiracetam	↓**	0.003383
9	Norquetiapine	↑**	0.003941
10	N,N-Diethyl-4-methyl-1-piperazinecarboxamide 4-oxide	↑**	0.006229
11	NP-012534	↑**	0.006938
12	Proline	↑**	0.008339
13	(2R)-1-[(1Z)-1-Octadecen-1-yloxy]-3-(phosphonooxy)-2-propanyl (4Z,7Z,10Z,13Z,16Z,19Z)-4,7,10,13,16,19-docosahexaenoate	↑**	0.009307
14	Sulfurous acid	↑*	0.016352
15	1-adamantyl(piperidino)methanone	↑*	0.017044
16	biperiden	↓*	0.017147
17	NSC 92778	↑*	0.028721
18	Arachidonoyl amide	↑*	0.030174
19	3,4-Dihydroxyphenylpropionic acid	↑*	0.039713
20	Lactamide	↑*	0.042595
21	VS-5584	↑*	0.049673

Compared to control group: ****$p < 0.0001$, ***$p < 0.001$, **$p < 0.01$, *$p < 0.05$.

Table S2. Significant metabolites and fold change analysis comparing control groups vs MPs exposure groups (100 μg/mL): MG1655 medium.

	Metabolites	MPs/Control (log2 FC)	*p*. value
1	N,N'-di[4-(2,6-dimethylmorpholino)phenyl]thiourea	↑****	3.86E-09
2	Lauryl sulfate	↑****	3.47E-08
3	Dinitrosopentamethylenetetramine	↑****	7.81E-06
4	UV9702000	↑****	7.81E-06
5	(2R)-2,3-Dihydroxypropanoic acid	↑****	1.76E-05
6	AY4375000	↓****	1.79E-05
7	2-Methylnaphthalene	↑****	9.20E-05
8	tyr-gln	↑***	0.000174
9	Aspartyl-L-proline	↑***	0.000213
10	Threonylglutamine	↑***	0.000213
11	N-(2-Aminopropanoyl)glutamine	↑***	0.000214
12	Pendimethalin	↑***	0.000218
13	4-(Dimethylamino)-N-[6-(hydroxyamino)-6-oxohexyl]benzamide	↑***	0.000283
14	arg-gln	↑***	0.000297
15	L-Glutaminyl-L-Arginine	↑***	0.000301
16	Leu-Val	↑***	0.000303
17	DL-Malic acid	↑***	0.000345
18	N(6),N(6)-Dimethyladenine	↑***	0.000397
19	5-(sec-butyl)-2-hydroxybenzaldehyde N-phenylhydrazone	↑***	0.000425
20	2-Amino-4-{6-[(3-methyl-2-buten-1-yl)amino]-3H-purin-3-yl}butanoic acid	↑***	0.000427
21	Leu-Leu	↑***	0.000474
22	2-AMINO-3,4-DIMETHYLIMIDAZO(4,5-F)QUINOLINE	↑***	0.000482
23	Daidzin	↑***	0.000486
24	Alanylclavam	↑***	0.000556
25	MFCD00025555	↑***	0.000699
26	N-(1H-Pyrrol-2-ylcarbonyl)glycine	↑***	0.000736
27	ciforadenant	↑***	0.000916
28	Pentosidine	↑**	0.001143
29	D-(+)-Malic acid	↑**	0.001195
30	OXOGESTONE PHENPROPIONATE	↑**	0.001325

Compared to control group: ****$p < 0.0001$, ***$p < 0.001$, **$p < 0.01$.

Table S3. Top 30 Significant metabolites and fold change analysis comparing control groups vs MPs exposure groups (100 μg/mL): Nissle1917 bacteria.

	Metabolites	MPs/Control (log2 FC)	*p*. value
1	N1-(2-methyl-4-nitrophenyl)acetamide	↑**	0.001317
2	acetylserine	↑**	0.001317
3	Valyl-4-hydroxyproline	↑**	0.001317
4	2,4-DIMETHYL-5-VINYLTHIAZOLE	↑**	0.001317
5	pyridoxal isonicotinoyl hydrazone	↑**	0.001317
6	Fumaric acid	↑**	0.001419
7	C4 H8 N2	↑**	0.001419
8	2-amino-2-deoxyisochorismic acid	↑**	0.001419
9	6-(1-Hydroxyethyl)-3-(hydroxymethyl)-2,7-dioxabicyclo[4.1.0]hept-3-en-5-one	↓**	0.001419
10	C3 H7 O7 P	↑**	0.001419
11	4-Aminobenzoic acid	↑**	0.001419
12	N-Acetylornithine	↑**	0.001470
13	5-[(1,3-Benzothiazol-2-ylmethyl)sulfanyl]-4-(2-furylmethyl)-4H-1,2,4-triazol-3-ol	↑**	0.001508
14	2-Aminoadipic acid	↓**	0.001529
15	REPAGLINIDE-D5	↑**	0.001529
16	DL-Mevalonic acid	↑**	0.001529
17	Adenine	↑**	0.001651
18	Demethon-S-methyl	↑**	0.001669
19	Lauryl sulfate	↑**	0.002030
20	norhaman	↓**	0.002030
21	Ser-Glu	↑**	0.002030
22	Thymidine 5'-monophosphate	↑**	0.002030
23	Phenethyl isothiocyanate	↑**	0.002030
24	Phenylisocyanate	↑**	0.002030
25	scyllo-Inosamine	↑**	0.002030
26	C9 H8 O3	↓**	0.002246
27	N-gondoylethanolamine	↓**	0.002504
28	3-Phosphoglyceric acid	↑**	0.002504
29	Phosphoenolpyruvic acid	↑**	0.002504
30	253GML9Q62	↑**	0.002504

Compared to control group: $^{**}p < 0.01$.

Table S4. Top 30 Significant metabolites and fold change analysis comparing control groups vs MPs exposure groups (100 μg/mL): Nissle1917 medium.

	Metabolites	MPs/Control (log2 FC)	*p*. value
1	N,N'-di[4-(2,6-dimethylmorpholino)phenyl]thiourea	↑***	0.000111
2	Gly-Leu	↑***	0.000125
3	FLK	↑***	0.000143
4	TLK	↑***	0.000313
5	8-Hydroxyhexadecanedioic acid	↑***	0.000313
6	NP-012534	↑***	0.000325
7	Lys-Lys	↑***	0.000405
8	HLK	↑***	0.000447
9	KMK	↑***	0.000447
10	AKK	↑***	0.000447
11	Lys-Arg	↑***	0.000455
12	KPK	↑***	0.000462
13	diphthinate	↑***	0.000462
14	RLK	↑***	0.000462
15	epsilon-(gamma-Glutamyl)-lysine	↑***	0.000517
16	thr-pro	↑***	0.000517
17	Leucylproline	↑***	0.000548
18	Acetohydroxamic acid	↑***	0.000686
19	9-Fluoro-16alpha-hydroxyandrost-4-ene-3,11,17-trione	↑***	0.000686
20	theodrenaline	↓***	0.000757
21	Lys-Glu	↑***	0.000757
22	Glycyl-L-leucine	↑***	0.000757
23	Lactamide	↑***	0.000850
24	tryprostatin A	↑***	0.000875
25	NKK	↑***	0.000981
26	RNK	↑**	0.001007
27	KNK	↑**	0.001028
28	Argininosuccinic acid	↓**	0.001028
29	THJ2201	↓**	0.001128
30	LNK	↑**	0.001128

Compared to control group: ***$p < 0.001$, **$p < 0.01$.

Table S5. Top 30 Significant metabolites and fold change analysis comparing control groups vs MPs exposure groups (100 μg/mL): *L. rhamnosus* bacteria.

	Metabolites	MPs/Control (log2 FC)	*p*. value
1	AGI-5198	↑****	5.52E-07
2	Aceglutamide	↑****	8.37E-06
3	H2 O4 S	↑****	5.17E-05
4	Angoline	↑***	0.000174
5	RAMIPRIL DIKETOPIPERAZINE ACID	↓***	0.000270
6	SECONAL	↑***	0.000631
7	H2 O4 S.1	↓***	0.000641
8	(12Z,15S,16S)-18-Bromo-15-hydroxy-12,16,17-octadecatrienoic acid	↑***	0.000641
9	4-HOBA	↑***	0.000641
10	2-{[(1-methyl-1H-pyrazol-5-yl)amino]methylene}malononitrile	↑***	0.000641
11	Ipatasertib	↑***	0.000641
12	BIX 02565	↑***	0.000641
13	N-(2,2,4,4-Tetramethyl-3-thietanyl)alaninamide	↑***	0.000641
14	S-Adenosylmethionine	↑***	0.000768
15	(3S)-3-(Pentanoyloxy)-4-(trimethylammonio)butanoate	↑***	0.000910
16	TIRON FREE ACID	↑**	0.001088
17	Perfluorobutane	↑**	0.001763
18	4-Hydroxyprolylthreonine	↓**	0.001848
19	NP-004768	↑**	0.002536
20	Spinosyn D	↓**	0.002679
21	glisoxepide	↑**	0.002679
22	Pimonidazole	↑**	0.002679
23	Rizatriptan	↑**	0.002847
24	NP-012534	↑**	0.002854
25	N8-Acetylspermidine	↑**	0.002862
26	Medrogestone	↓**	0.002862
27	N1,N12-Diacetylspermine	↑**	0.002983
28	Acevaltrate	↑**	0.003036
29	WLK	↑**	0.003036
30	Pitavastatin	↑**	0.003144

Compared to control group: ****$p < 0.0001$, ***$p < 0.001$, **$p < 0.01$.

Table S6. Significant metabolites and fold change analysis comparing control groups vs MPs exposure groups (100 μg/mL): *L. rhamnosus* medium.

	Metabolites	MPs/Control (log2 FC)	*p*. value
1	NP-012534	↑****	2.36E-06
2	C14 Ceramide	↑**	0.003838
3	5-[6-(3-Methoxy-3-oxetanyl)-7-methyl-4-(4-morpholinyl)thieno[3,2-d]pyrimidin-2-yl]-2-pyrimidinamine	↑**	0.004797
4	Tampramine	↓**	0.009419
5	GPi 819	↓*	0.012904
6	glaucarubolone	↑*	0.013596
7	DL-4-Hydroxyphenyllactic acid	↑*	0.014067
8	3,4-Methylenedioxy-N-benzylcathinone	↑*	0.018046
9	Ezutromid	↓*	0.023126
10	Methyl 3-{1-[2,4-dihydroxy-6-methoxy-3-(3-phenylpropanoyl)phenyl]-3-methyl-2-buten-1-yl}-4-hydroxybenzoate	↑*	0.026508
11	Tomelukast	↓*	0.026752
12	aticaprant	↓*	0.032648
13	Amide C22	↑*	0.034892
14	Malonyl-L-carnitine	↑*	0.038190
15	2-(5-Methyl-2-furyl)-3-piperidinol	↓*	0.038457
16	Bayer E 39 Soluble	↑*	0.040338
17	glyceraldehyde 3-phosphate	↓*	0.045141

Compared to control group: ****$p < 0.0001$, **$p < 0.01$, *$p < 0.05$.

Table S7. Top 30 Significant metabolites and fold change analysis comparing control groups vs MPs exposure groups (100 μg/mL): gut bacteria extracted from C57BL/6 mouse feces.

	Metabolites	MPs/Control (log2 FC)	*p*. value
1	DL-Alanyl-DL-leucine	↓****	1.08E-07
2	Dodecyl sulfate	↑***	1.61E-06
3	Elaeokanine C	↑***	2.03E-06
4	N-Decanoylglycine	↑***	2.03E-06
5	9-Oxononanoic acid	↓***	3.78E-06
6	Gabapentin	↓***	3.55E-06
7	Juniperic acid	↑***	3.16E-06
8	1,3,7-Octanetriol	↓***	5.45E-06
9	1,2,4-Butanetriol	↓***	5.51E-06
10	Aceglatone	↓***	4.61E-06
11	Ebelactone B	↑***	6.92E-06
12	benzal chloride	↓***	6.91E-06
13	3-Thiomorpholinecarboxylic acid	↑***	1.13E-05
14	1-Palmitoyl-2-Oleoyl-sn-glycero-3-PA	↑***	1.56E-05
15	Glycylglutamine	↓***	1.73E-05
16	Sulfamic acid	↓***	1.89E-05
17	Indigo	↑***	2.31E-05
18	O-ureido-D-serine	↓**	3.27E-05
19	Linalyl propionate	↓**	3.52E-05
20	3-oxopalmitic acid	↑**	3.67E-05
21	2-decene-4,6,8-triyn-1-al	↓**	3.98E-05
22	16-Heptadecene-1,2,4-triol	↑**	5.88E-05
23	Phosphoarginine	↓**	0.000106
24	O-(17-carboxyheptadecanoyl)carnitine	↑**	0.000119
25	Latanoprost	↑**	0.000185
26	Sulfuric acid	↑**	0.000192
27	11-Nitro-1-undecene	↓**	0.000200
28	Hydroxynervonic acid	↑**	0.000221
29	9-Hexadecenoylcarnitine	↑**	0.000238
30	15-Methylheptadecanamide	↑**	0.000294

Compared to control group: ****$p < 0.0001$, ***$p < 0.001$, **$p < 0.01$.

Table S8. Significant metabolites and fold change analysis comparing control groups vs MPs exposure groups (100 μg/mL): medium samples for the gut bacteria extracted from C57BL/6 mouse feces.

	Metabolites	MPs/Control (log2 FC)	*p*. value
1	Picfeltarraenin IA	↑****	4.90E-10
2	laurilsulfate	↑**	9.01E-06
3	Cilnidipine	↑*	6.42E-05
4	EADB-FUBINACA	↑*	8.30E-05
5	Bekanamycin	↑*	0.000219
6	pNPS-DHA	↑*	0.000340

Compared to control group: ****$p < 0.0001$, **$p < 0.01$, *$p < 0.05$.
